# The effect of green kiwifruit on gas transit and tolerance in healthy humans

**DOI:** 10.1111/nmo.13874

**Published:** 2020-05-20

**Authors:** Noemi Caballero, Bouchra Benslaiman, Juliet Ansell, Jordi Serra

**Affiliations:** ^1^ Motility and Functional Gut Disorders Unit Centro de Investigación Biomédica en Red de Enfermedades Hepáticas y Digestivas (CIBERehd) University Hospital Germans Trias i Pujol Badalona Spain; ^2^ Department of Medicine Autonomous University of Barcelona Badalona Spain; ^3^ Zespri International Limited Mt Maunganui New Zealand

**Keywords:** bloating, constipation, intestinal gas, kiwifruit

## Abstract

**Background:**

Green kiwifruit is a fiber‐rich fruit that has been shown effective for treatment of constipation. However, fermentation of fibers by colonic bacteria may worsen commonly associated gas‐related abdominal symptoms. Aim: To determine the effect of green kiwifruit on transit and tolerance to intestinal gas in humans.

**Methods:**

In 11 healthy individuals, two gas challenge tests were performed (a) after 2 weeks on a low‐flatulogenic diet and daily intake of 2 green kiwifruits and (b) after 2 weeks on a similar diet without intake of kiwifruits. The gas challenge test consisted in continuous infusion of a mixture of gases into the jejunum at 12 mL/min for 2 hours while measuring rectal gas evacuation, abdominal symptoms, and abdominal distension. During the 2 weeks prior to each gas challenge test (on‐kiwifruit and off‐kiwifruit), the number and consistency of stools, and abdominal symptoms were registered.

**Key Results:**

Intake of kiwifruits was associated with more bowel movements per day (1.8 ± 0.1 vs 1.5 ± 0.1 off‐kiwifruit; *P* = .001) and somewhat looser stools (Bristol score 3.3 ± 0.2 vs 2.8 ± 0.1 off‐kiwifruit; *P* = .072) without relevant abdominal symptoms. Gas infusion produced similar gas evacuation (1238 ± 254 mL and 1172 ± 290 mL; *P* = .4355), perception of symptoms (score 1.2 ± 0.2 and 1.3 ± 0.3; *P* = .2367), and abdominal distension (17 ± 7 mm and 17 ± 6 mm; *P* = .4704) while on‐kiwifruit or off‐kiwifruit.

**Conclusions and Inferences:**

In healthy subjects, green kiwifruit increases stool frequency without relevant effects on intestinal gas transit and tolerance. If confirmed in patients, these fruits may provide a natural and well‐tolerated treatment alternative for constipation.

## Graphical abstract

In a cross‐over, randomized study in healthy subjects, intake of the fibre‐rich green kiwifruit increased bowel movements without effects on gas transit and tolerance in response to a gas challenge test. If confirmed in patients, these fruits may provide a natural and well tolerated treatment alternative for constipation.

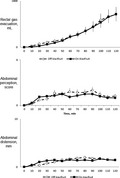

**Key Points**

- Green kiwifruit is a fiber‐rich fruit that has been shown to be effective for treatment of constipation in humans. However, fermentation of fibers by colonic bacteria may worsen commonly associated gas‐related abdominal symptoms.
- In a cross‐over, randomized study in healthy subjects, intake of green kiwifruit increased bowel movements without effects on gas transit and tolerance in response to a gas challenge test.
- In healthy subjects, the fiber‐rich green kiwifruit increases stool frequency without relevant effects on intestinal gas transit and tolerance. If confirmed in patients, these fruits may provide a natural and well‐tolerated treatment alternative for constipation.

## 1 INTRODUCTION

Kiwifruit is a fruit rich in fibers, C, E, and K vitamins, folate, potassium, and other phytochemicals that has been shown to have several beneficial effects on gastrointestinal health.^3^, ^12^ Specifically, kiwifruit has been shown to accelerate gastric and colonic transit, soften the stools, and to improve constipation, an effect that can be partially mediated by the high fiber contents of kiwifruit.^3^, ^4^, ^5^ Patients with constipation and other related functional gut disorders like constipation‐predominant irritable bowel syndrome often refer gas‐related abdominal complains, like bloating and abdominal distension, that in many cases are referred as their most bothersome symptoms.^6^, ^7^ Fibers have a high potential to produce intestinal gas by increasing bacterial fermentation in the colon, and the development of gas‐related abdominal symptoms may be a limiting factor for prescription and intake of fibers and some laxatives like lactulose that are fermented by colonic bacteria.^8^, ^9^, ^10^

Kiwifruits contain 1.4‐3.0 g. of dietary fiber per 100 g. of fruit. One third soluble fiber, mainly pectic polysaccharides, that when reaching the colon should be fermented by colonic bacteria and produce gases and short‐chain fatty acids, and two thirds insoluble fiber, mainly cellulose and hemicellulose which should not produce any fermentation when reaching the colon.^1^, ^2^, ^11^ The effect of kiwifruits on gas transit and evacuation is uncertain. Previous studies from other laboratories have shown that psyllium and high‐calorie meals delay gas transit and evacuation.^12^, ^13^ In addition, trapping of gas by viscous material, as has been described for water after intake of a similar quantity of kiwifruit,^14^ could delay gas transit and produce gas‐related abdominal symptoms. On the other hand, a prokinetic effect of kiwifruit^5^ could accelerate transit and removal of intestinal gas, and thereby prevent gas‐related abdominal symptoms while improving constipation.

The aim of the present study was to determine the effect of green kiwifruits on transit and tolerance to intestinal gas in healthy subjects. Using a previously developed and validated method of intestinal gas challenge,^15^, ^16^, ^17^, ^18^ we developed a randomized, cross‐over study comparing, on separate days, the responses to a gas challenge test performed after a 14‐day low‐flatulogenic diet including intake of two green kiwifruits per day, or after a 14‐day low‐flatulogenic diet without intake of kiwifruit. Due to the rapid normal transit of gas in healthy subjects, a concomitant lipid infusion into the proximal duodenum, mimicking the physiological postprandial caloric load, was perfused during gas infusion to delay intestinal transit of gas, as used in previous studies.^16^, ^19^, ^20^

## 2 MATERIAL AND METHODS

### 2.1 Participants

Eleven healthy individuals (8 women and 3 men; age range 18‐23 years) without gastrointestinal symptoms participated in the study. Subjects completed a pre‐entry questionnaire to determine the absence of gastrointestinal symptoms, including abdominal pain, dyspepsia, bloating, constipation, diarrhea, feeling of excessive abdominal gas, excessive gas evacuation, or belching. The protocol for the study had been previously approved by the Ethics Committee of the University Hospital Germans Trias i Pujol, and all subjects gave written informed consent before participating in the study.

### 2.2 Intervention period

Two weeks before the gas challenge test (see below), participants were instructed to follow a low‐flatulogenic diet excluding legumes, vegetables, garlic, onion, cucumber, nuts, cereals, whole‐meal bread, and fizzy drinks. In random order, participants were assigned to eat 2 green kiwifruits without skins every morning (Zespri International, approximate weight 150 g per kiwifruit), or to follow the same low‐flatulogenic diet but with no intake of kiwifruits.

During the 2‐week intervention periods, participants filled a diary recording in separate questionnaires the number and type, according to the Bristol stool form scale, of bowel movements, the intensity of abdominal pain and the intensity of abdominal bloating, both using 0‐3 score scales were 0 was no sensation, 1 mild sensation, 2 moderate sensation and 3 severe sensation. In addition, the number of kiwifruit ingested was registered.

### 2.3 Gas challenge test

The gas challenge test was performed using a methodology that has been extensively used and validated in detail.^15^, ^16^, ^17^, ^18^, ^21^, ^22^, ^23^, ^24^

#### 2.3.1 Jejunal gas infusion

Gas was continuously infused into the proximal jejunum at 12 mL/min via an oro‐intestinal polyvinyl tube (3.2‐mm external diameter; 1.6‐mm internal diameter) connected to a volumetric pump (GIP‐3000 Infusion Pump, Soifer Solucions SL). The gas mixture infused contained 88% nitrogen, 6.5% carbon dioxide, and 5.5% oxygen mimicking the partial pressures of venous blood gases to minimize diffusion across the intestine‐blood barrier.

#### 2.3.2 Measurement of rectal gas evacuation

Intestinal gas evacuation was collected via an intrarectal Foley catheter (20 French; Bard) with the balloon inflated with 5 mL of water. The catheter was connected to a leak‐proof, low‐resistance collection line, using a barostat, and the volume continuously recorded on a personal computer, as previously described.

#### 2.3.3 Lipid infusion

A mixture of lipids (Intralipid^®^ 20%; Pharmacia & Upjohn, St Cugat del Vallés) diluted in saline was infused into the duodenum at 1 mL/min (1 Kcal/min) using a volumetric pump (Alaris GH Plus Guardrails; CareFusion). Lipids were infused via a 1.2 mm ID channel added to the intestinal tube assembly, with its distal port located in the duodenum 15 cm orad to the gas infusion site.

#### 2.3.4 Intestinal gas tolerance

Conscious perception was measured at 10‐minute intervals by means of four graphic rating scales, each graded from 0 (no perception) to 6 (painful sensation), specifically for scoring four possible types of abdominal sensations: (a) pressure/bloating, (b) cramp/rumbling sensation, (c) puncture/stinging sensation, and (d) other type of sensation (to be specified), respectively. The questionnaire included an additional tic box (YES/NO) to signal belching. The location of the perceived sensations was marked on an abdominal diagram divided into 9 regions corresponding to epigastrium, periumbilical area, hypogastrium, both hypochondria, flanks, and iliac fossae. Participants were instructed to report the sensations perceived over the preceding 10‐minute period in the scales.

#### 2.3.5 Abdominal distension

Once the subjects were positioned in bed (Section 2.3.6), a non‐stretch 48‐mm wide belt with a metric tape measure was adjusted around the abdomen over the umbilicus by means of two elastic bands. Girth measurements were taken at 10‐minute intervals, while the subjects were breathing relaxedly, as the average of inspiratory and expiratory determinations over three consecutive respiratory excursions.

#### 2.3.6 Procedure

After the 2‐week intervention period (see above), participants were instructed to have a light dinner the night before the gas challenge test, that consisted of meat, fish, eggs, rice, pasta, and/or white bread, but avoiding dairy products, salad, fruit, and alcoholic beverages. On the day of the study, participants were orally intubated after an 8‐hour fast. No kiwifruit was eaten the day of the study. The intestinal tube assembly was positioned under fluoroscopic control with the gas infusion port 5 cm caudad to the angle of Treitz. The rectal cannula was introduced, and the subjects were then placed supine in bed at an angle of 30° to the horizontal. The studies were conducted in a quiet, isolated room. The tubes were connected to the infusion and collection systems, and gas transit was continuously recorded while perception and abdominal girth were measured at 10‐minute intervals.

### 2.4 Experimental design

**Figure 1:**
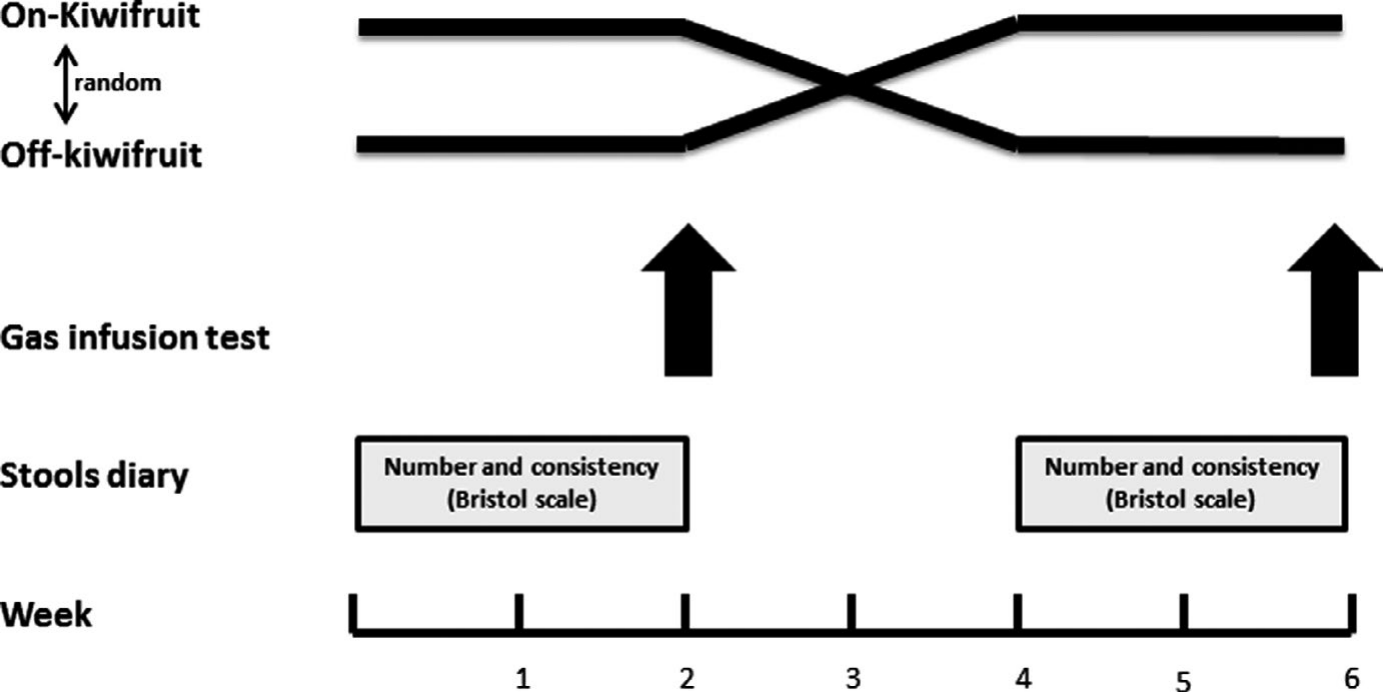
Experimental design. In each participant, two gas challenge tests were performed after 2 wk on‐kiwifruit and off‐kiwifruit using a randomized cross‐over design. While on‐kiwifruit, last kiwifruit intake occurred 24 h, before the gas challenge test

A two‐period, cross‐over, randomized, single‐blind study was designed to determine the effect of green kiwifruit intake on gas transit and tolerance (Figure 1). In each participant, two gas challenge tests were performed with a 4‐week interval. Gas challenge tests were preceded by a 2‐week intervention period with the subjects on a low‐flatulogenic diet with or without daily intake of 2 green kiwifruits. After a 2‐week washout period, a new 2‐week intervention period started and the second gas challenge test was performed. The gas challenge tests were performed with the investigator blind for the assigned intervention.

### 2.5 Data analysis

In each subject, the number and type of bowel movements, and the intensity of perception during each 2‐week intervention periods were registered for future comparisons. During each gas challenge test, intestinal gas transit was evaluated by the volume of gas retained within the gut (volume infused minus volume collected) at different intervals during the study. Perception was measured by the score rated in the scales; the highest score (the most severe sensation independently of the type), instead of the mean or cumulative scores, was computed for comparisons. In each subject, we also counted the number of times each abdominal sensation was scored in the repeat measurements during the study to calculate the frequency (as percent distribution) of each specific sensation. In the anatomical questionnaire, we calculated the percentage of sensations referred over each abdominal region, as well as the percentage referred over more than one region. Changes in abdominal girth during the study were referenced to girth measurement at the start of the study, that is before gas infusion was started.

### 2.6 Statistical analysis

Based on previous studies performed using an identical methodology in healthy subjects,^15^, ^16^, ^17^ we calculated that a sample size of 10 subjects per group could discriminate differences in gas retention with a power of 80% and an alpha error of 5%.

In each treatment period, mean values (±SE) of the parameters measured were calculated. The Kolmogorov‐Smirnov test was used to check the normality of data distribution. Comparisons of parametric, normally distributed data were performed by the Student *t* test for paired data. Non‐parametric data were compared using the Wilcoxon signed‐rank test.

## 3 RESULTS

### 3.1 General aspects

Eleven healthy subjects (8 women and 3 men; age range 18‐23 yrs) were included in the study. All the subjects completed the 2‐week intervention period uneventfully. All subjects scored intake of two kiwifruits, or 0 kiwifruits, as corresponded, during the 14 days prior to each gas challenge test. All participants but one completed the 2 gas challenge test procedures as planned, so finally, the results of 10 healthy subjects who completed the study could be analyzed. The reason for withdrawal of one healthy subject was a technical problem with the gas‐recovery system that did not allow measurement of gas evacuation during the second gas transit procedure. All the remaining 10 participants completed the study without remarkable events.

### 3.2 Effects of kiwifruit on abdominal symptoms, stool consistency, and frequency

**Figure 2:**
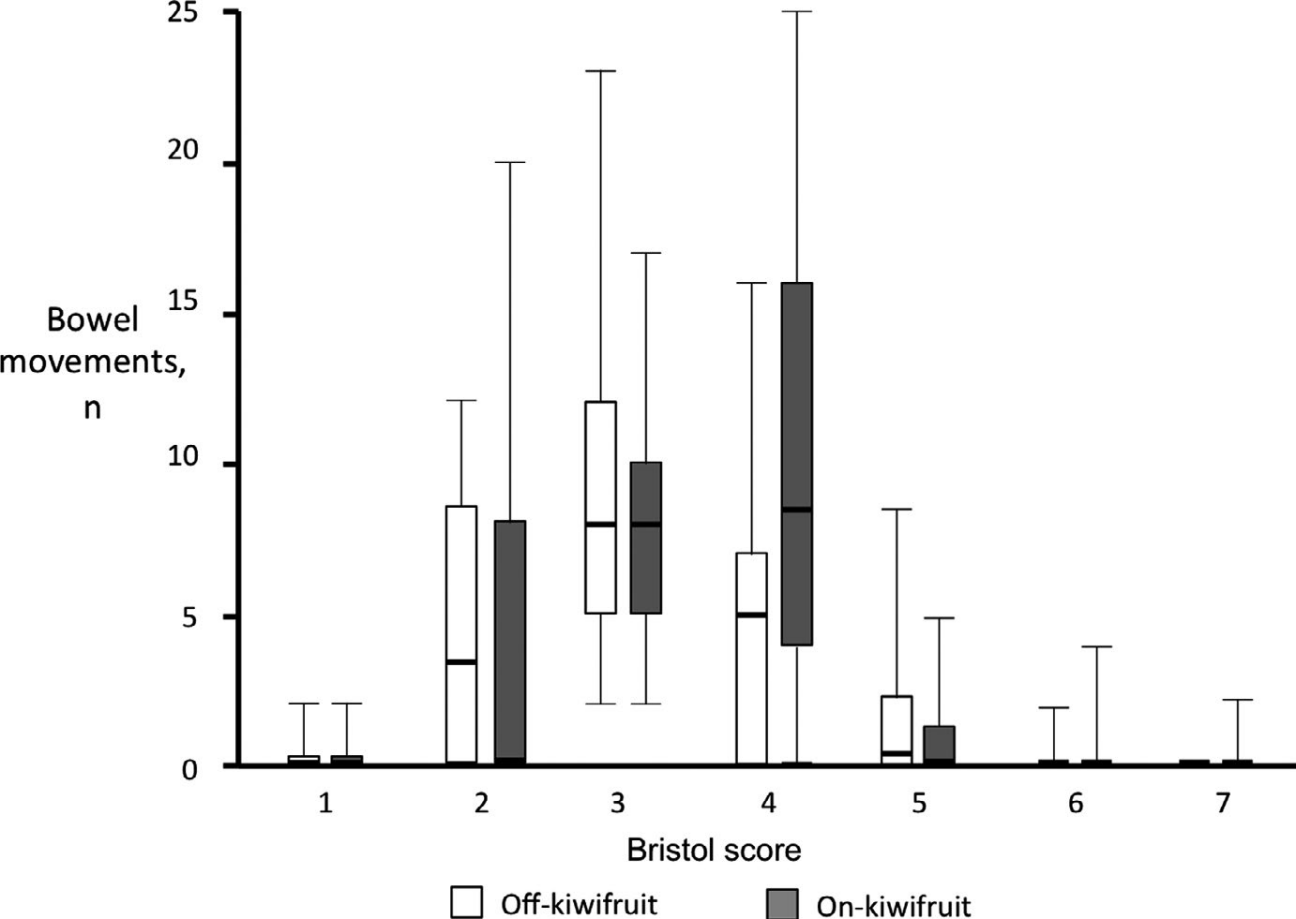
Form of stools according to the Bristol Stool Form Scale during 2‐wk low flatulogenic diet on‐kiwifruit (intake of 2 pieces of green kiwifruit per day) and off‐kiwifruit. Note that the predominant stool form moved from type 3 while off‐kiwifruit to type 4 on‐kiwifruit, reflecting a looser consistency of stools, but within the normal range of stool form

Kiwifruit intake during the 2 weeks preceding the gas transit procedure was well‐tolerated by all the subjects and induced neither abdominal pain (score 0.1 ± 0.1) nor bloating (score 0.1 ± 0.0). Similarly, during the 2 weeks off‐kiwifruit no relevant abdominal symptoms were rated (pain score 0.0 ± 0.0; bloating score 0.1 ± 0.0). The number of bowel movements was somewhat, but significantly greater when participants were on a diet including intake of 2 green kiwifruits per day (1.8 ± 0.1 stools/d), than during the 2 weeks off‐kiwifruit (1.5 ± 0.1 stools/d; *P* = .001). The consistency of the stools measured by the Bristol Stool Form Scale tended to be softer while on‐kiwifruit (Figure 2). The predominant stool form off‐kiwifruit was Bristol 3, whereas the predominant stool form on‐kiwifruit was Bristol 4, but these differences were minor and did not reach statistical significance (*P* = .068). Hence, most stools lied within the normality range (Bristol score 3‐5), and only one subject while on‐kiwifruit and one subject while off‐kiwifruit referred some loose stools (Bristol 6 or 7) during the intervention period (*P* = .655).

### 3.3 Effect of kiwifruit on gas transit during the gas challenge test

**Figure 3:**
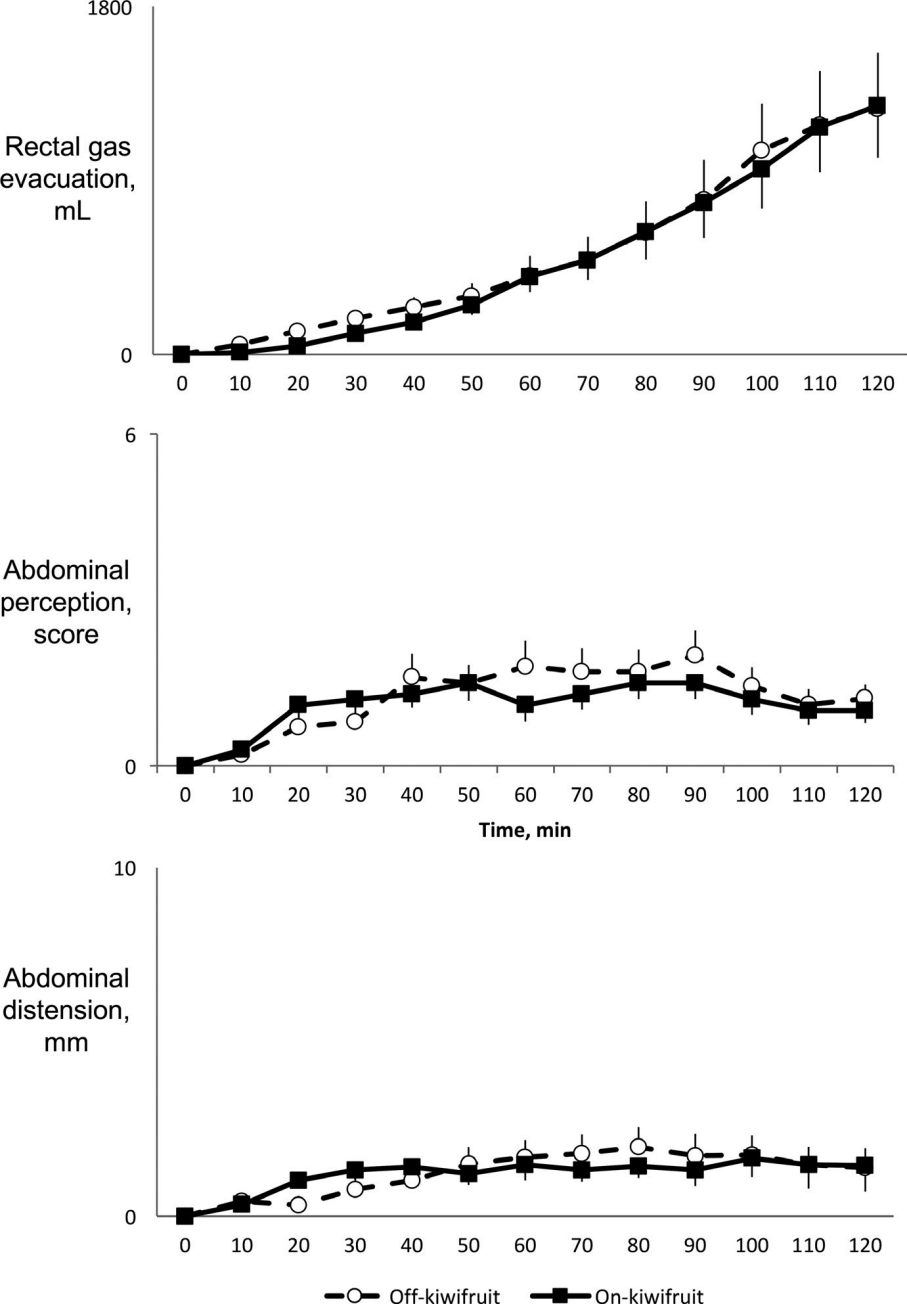
Responses to a gas challenge test (infusion of gas into the jejunum al 12 mL/min for 120 min) performed after 2 wk on a low‐flatulogenic diet on‐kiwifruit (intake of 2 pieces of green kiwifruit per day) and off‐kiwifruit. Observe that there were no differences in rectal gas evacuation, perception of abdominal symptoms or objective abdominal distension in the studies performed on‐kiwifruit and off‐kiwifruit

Gas infusion produced a continuous evacuation of gas from the rectum, without any differences whether gas infusion was performed after 2 weeks daily kiwifruit intake or not (Figure 3). Hence, after an initial lag time that resulted in a maximal gas retention of 340 ± 122 and 334 ± 104 mL after 70 minutes of gas infusion (studies off‐kiwifruit and on‐kiwifruit, respectively; *P* = .484), at the end of the infusion period, participants had retained 206 ± 280 mL after 2 weeks off‐kiwifruit, and 123 ± 250 mL of gas when gas infusion was performed after 2 weeks on‐kiwifruit (*P* = .417).

### 3.4 Effect of kiwifruit on abdominal symptoms during the gas challenge test

Gas infusion was well‐tolerated by all the subjects and produced a mild increment in abdominal perception during the first 60 minutes of infusion, that was then maintained until the end of the study, without differences whether the gas transit studies were performed off‐kiwifruit or on‐kiwifruit (Figure 3). Hence, mean perception score was 1.3 ± 0.8 off‐kiwifruit and 1.2 ± 0.2 on‐kiwifruit (*P* = .4733). When individual data were analyzed, no subject referred discomfort (score 5) and only 3 subjects developed moderate perception (score 3 or 4) at the end of the gas challenge test in the studies performed off‐kiwifruit.

The predominant symptoms referred during the gas challenge test were pressure/bloating and cramp/rumbling sensation, mainly referred over the abdominal midline, without differences whether the test was performed on‐kiwifruit or off‐kiwifruit (data not shown).

### 3.5 Effect of kiwifruit on objective abdominal distension during the gas challenge test

Before the start of the gas infusion procedure, at arrival to the laboratory, we found no differences in abdominal girth whether the subjects had been eating 2 green kiwifruits per day during 14 days (abdominal girth 79.2 ± 3.7 cm) or were not eating kiwifruit (abdominal girth 79,7 ± 3.7 cm: *P* = .3058). Gas infusion was associated with a minor increment in abdominal girth during the first 60 minutes of gas infusion, that was then maintained until the end of the study, without differences whether the gas transit studies were performed on‐kiwifruit or off‐kiwifruit (Figure 3). Hence, final abdominal distension was 1.7 ± 0.6 cm off‐kiwifruit and 1.7 ± 0.7 on‐kiwifruit (*P* = .4704).

## 4 DISCUSSION

In the present study performed in healthy subjects, we have shown that daily intake of two green kiwifruits per day increases stool frequency and soften the stools without inducing gas‐related abdominal symptoms, without increasing rectal gas evacuation, and without effects on gas transit and tolerance to intestinal gas loads.

In patients with constipation, increased dietary fibers are considered first‐line treatment in most of the published guidelines on chronic constipation and constipation associated with irritable bowel syndrome.^25^, ^26^, ^27^, ^28^, ^29^ However, patients with constipation often complain of associated symptoms like bloating and abdominal distension, which may worsen after fiber consumption due to the increased intestinal water and gas volumes induced by fermentation of the complex polysaccharides that are components of natural fibers.^10^, ^30^ Already in 1969, Steggerda described an increment in flatus production following consumption of pinto beans,^31^ and several studies published the following years confirmed these observations.^32^, ^33^ In patients with functional gut disorders, increased colonic contents may induce bloating and distension by several mechanisms, including visceral hypersensitivity,^34^ altered transit and evacuation of gas,^18^ and altered viscero‐somatic reflex responses with abdomino‐phrenic dysfunction.^35^

Among the aliments that may be useful for the first‐line management of constipation, green kiwifruit is a fiber‐rich fruit that has been shown to relief constipation in different clinical trials.^3^, ^4^ As most natural aliments, kiwifruit contains a mixture of soluble and non‐soluble fibers. Specifically, in the present study, the subjects consumed 2 green kiwifruits per day, a number of fruits that have been shown to improve constipation in clinical trials, containing 9 g of dietary fibers. Despite its potential gas production, during the 2 weeks kiwifruit intake participants did not complain of any gas‐related abdominal symptoms, and when a gas infusion test was performed, the volume of gas expelled was similar whether the subject had been on‐kiwifruit or off‐kiwifruit during the 2 weeks prior the gas infusion test. Previous studies have shown that the increase in gas production induced by fibers depends on the type of fibers ingested. Hence, Levitt et al^36^ compared the effect of lactulose, the poorly fermentable fiber psyllium, and the non‐fermentable fiber methylcellulose on flatus production in healthy subjects, and found that whereas lactulose increased flatus production significantly, none of the fibers tested induced any increment in flatus production. Likewise, Gonlachanvit et al^1^ using a similar gas infusion technique as we used in the present study found that psyllium consumption delayed gas transit without any associated increment in gas evacuation. In a randomized clinical trial comparing the effect of psyllium vs a mixture of soluble and insoluble fibers in patients with constipation, gas‐related abdominal symptoms improved most when the mixed fibers were consumed.^37^ Hence, these data may suggest that a delay in gas transit induced by some fibers could worsen abdominal symptoms like bloating. In our study using kiwifruit, a mixture of soluble and insoluble fibers, no delay in gaseous transit time was observed after kiwifruit ingestion. Previous studies using radiopaque markers have shown that green kiwifruit accelerates whole gut transit time in patients with constipation and constipation‐predominant irritable bowel syndrome.^3^, ^4^, ^5^ In a recent study from Nottingham using MRI in healthy subjects, the authors found that ingestion of two green kiwifruits per day, the same intake as we used in the present study, produced an increment in water content in the small bowel and the ascending colon that could account for the increment in stool frequency and looser stool consistency observed.^14^ However, in this study, the authors found the increment in colonic contents induced by kiwifruit, smaller than the increment induced by intake of psyllium in previous studies. Hence, kiwifruit induced a rise in the colon volume by 16% whereas psyllium produced a 50% increment. The authors conclude that kiwifruits have prokinetic effects in addition to water trapping, leading to less colonic distension. Taking together the results of these studies, it seems plausible that a prokinetic effect of kiwifruit, maybe associated with the mixed type of fiber contents, could lead to lower water and gas retention into the colon, and result in lower bloating and abdominal distension than other dietary fibers or fermentable carbohydrates.

In addition to flatus production, another mechanism that reduces intestinal gases is diffusion of gases to the bloodstream.^8^ Bacterial fermentation produces different gases, mainly H_2_, CH_4,_ and CO_2_. When in the intestinal lumen, these gases are partially absorbed to the bloodstream. Diffusion of gases depends on the pressure gradient across the intestinal/blood barrier and the solubility in lipids of each specific gas.^9^, ^38^ By contrast to nitrogen, which has a poor diffusivity, gases produced during fermentation diffuse easily to the bloodstream. It has been calculated that about 2/3 of the H_2_ produced during bacterial fermentation is absorbed under basal conditions.^32^ Hence, a part of gases produced after kiwifruit intake can also be cleared from the intestine by this mechanism. However, gas infusion as was performed in the present study produces a washout effect of intestinal gases that should lead to increased rectal gas evacuation during the gas infusion procedure if a significant volume of gas was produced during kiwifruit intake.^17^, ^33^ Hence, even though no measurement of exhaled gas was performed in our study, our results suggest that the volume of intestinal gases produced during kiwifruit intake is small, and of low clinical significance for development of gas‐related symptoms.

The data of our study apply for healthy subjects with normal bowel habits. Further studies are necessary to determine the effects of kiwifruits on transit and tolerance of intestinal gas in patients with constipation. Previous studies have shown that patients with irritable bowel syndrome and abdominal bloating have a delayed transit of intestinal gas that is associated with abdominal symptoms.^16^, ^18^ In addition, these patients may be more sensitive to distension than healthy controls and so may tolerate kiwifruit less well as is true of fructose and fructans.^39^ Hence, we cannot ascertain that even small volumes of gas produced during kiwifruit intake could lead to gas retention and gas‐related abdominal symptoms in these specific patients.

In the present study, the quantity of fermentable fiber contained in the kiwifruits administered during the study was smaller than the quantity of fibers administered in other studies using for example psyllium. This relatively small volume was chosen because this is the recommended for treatment of constipation, and reflects the clinical consequences of treatment with kiwifruit. But we cannot exclude that intake of greater number of kiwifruits, and thereby a greater quantity of fibers, could produce different gas‐related responses, as previously described for other type of fibers.^1^

The present study was powered to assess the effect of kiwifruit on gas transit and evacuation in response to a gas challenge test, but not to assess the effect of green kiwifruit on constipation or stool consistency. However, and despite the low number of participants included, we could see a small but significant increment in the number of stools, and a tendency towards looser stools while participants were on‐kiwifruit, in accordance with the previously reported effects of green kiwifruits in patients with constipation.^3^, ^4^, ^5^ To note, in our cohort of healthy subjects, these effects were small and did not lead to diarrhea or an abnormal increment of bowel movements.

In conclusion, our data have shown that in healthy subjects, a daily intake of 2 green kiwifruits per day increases stool frequency without relevant effects on intestinal gas transit and tolerance. Though our study did not specifically address the clinical effectiveness of kiwifruit for patients with constipation, our data add to a growing body of data that these fruits may be a natural and well‐tolerated first‐line treatment for patients with constipation.

## CONFLICT OF INTEREST

This work has been performed as part of the doctoral thesis in medicine of Dr Caballero at the Autonomous University of Barcelona. Dr Serra has received research funds from Salvat, Zespri, and Bayer and acted as consulter/speaker for AB‐biotics, Allergan, Bayer, Norgine, Cassen‐Recordati, Zespri, and Reckitt Benkiser. Juliet Ansell is enployed by Zespri. Noemi Caballero and Bouchra Benslaiman have nothing to disclouse.

## AUTHOR CONTRIBUTIONS

NC involved in acquisition of data, analysis and interpretation of data, drafting of the manuscript, and statistical analysis; BB was Nurse coordinator and involved in recruitment of volunteers and acquisition of data; JA involved in study concept and design, obtained funding; JS involved in study concept and design, analysis and interpretation of data, critical revision of the manuscript for important intellectual content, statistical analysis, and obtained funding.
